# A feedback journey: employing a constructivist approach to the development of feedback literacy among health professional learners

**DOI:** 10.1186/s12909-021-02914-2

**Published:** 2021-09-10

**Authors:** Anne O’Connor, Arlene McCurtin

**Affiliations:** 1 School of Allied Health, University of Limerick, Limerick, Ireland; 2 Health Research Institute, University of Limerick, Limerick, Ireland; 3 Health Implementation Science & Technology Research Group, University of Limerick, Limerick, Ireland

**Keywords:** Feedback literacy, Health professions, Academic advisor, Critical thinking, Reflective practice, Lifelong learner, Co-construction

## Abstract

**Background:**

Feedback, if effectively provided by the teacher and utilised by the learner, enables improvement in academic performance. It is clear from current literature that the provision of feedback by teachers is not sufficient on its own to guarantee improvements as early university entrants may not be sufficiently equipped to effectively engage with feedback. Nonetheless, it is critical for health professional students to develop feedback literacy early, in order to prepare them for a professional career of lifelong learning and critical thinking. The overarching aim of this study was to identify a feasible, sustainable approach to improve feedback literacy among students on pre-qualifying health professional programmes.

**Methods:**

The study was divided into two phases. A mixed-methods approach grounded in constructivism was employed. Participants included teachers and learners from the School of Allied Health at X University, and two internationally acclaimed educationalists. In phase 1, first year students were encouraged to use an established online platform to upload modular feedback and develop personal learning action plans aimed at improving academic performance. A follow-up survey highlighted poor engagement with this method. Thus, the second phase focused on the co-construction of a suite of modules to develop these skills, supported by academic staff. Interviews were conducted with participants to review and refine this initiative.

**Results:**

Learners’ engagement with the first phase of the study was poor. Thus, the second phase provided all stakeholders with the opportunity to feed into the development of a suite of modules, designed to encourage teachers and learners to work in partnership to nurture these skills. All stakeholder groups reported short- and long-term benefits with this approach, but also highlighted challenges towards its implementation.

**Conclusion:**

The development of feedback literacy among health professional learners is essential. The transferability of such skills has been highlighted in the literature and by all stakeholder groups involved in this study. Finding a balance between introducing these skills at a time early enough to highlight their importance among university entrants is challenging. Further balance must be achieved between the workload required to achieve these skills and current programme demands for both teachers and learners.

**Supplementary Information:**

The online version contains supplementary material available at 10.1186/s12909-021-02914-2.

## Background

In many under-graduate and post-graduate health profession qualification programmes, clinical learning in the workplace is usually preceded by a substantial period of academic study. Effort is typically expended by teachers in preparing learners for clinical learning through teaching, assessment and feedback provision. Feedback, if effectively provided by the teacher and utilised by the student, enables the learner to improve subsequent performance on both academic and clinical assessments. Thus, effective feedback systems support learners to understand where additional effort is needed and how to achieve intended learning goals [1, 2]. Recommendations for enhancing feedback practices to improve feedback literacy pervade the literature and include the use of reflective practice, the development of self-regulation and the provision of feedback which is easily understood and directly applicable to future work [3–6]. Feedback literacy, according to Carless and Boud [7] is, the ability to appreciate and understand the feedback provided, self-evaluate one’s own work, attend to the feelings that feedback invokes and be able to act upon feedback provided. Thus, feedback literacy is critical to learning.

It is clear from current literature that the provision of feedback by teachers to learners is not sufficient on its own to guarantee improvements in academic and or clinical performance. For feedback to benefit the learner and improve performance, it must be tailored for use by the student for whom it is intended and, it must be utilized by that student. However, Barton et al. [8] note in their study promoting feedback literacy, that teachers can spend considerable time “crafting feedback” which in turn is not read by the learner thus rendering many feedback processes futile and time-consuming exercises. Thus, it is clear from this and other studies that learners do not always appreciate teachers’ time and effort in feedback provision often perceiving little value in its content [1, 9, 10]. Underuse of feedback may be due to the learner’s inability to understand how such feedback can assist in improving performance during their programme of study and onward into future learning contexts [9, 10]. Further, learners tend to value grades more than learning, with society and educational practices socialising students into being grade-oriented early in life [11, 12]. However, in order to fully appreciate the learning journey and the transferable skills obtained therein, learners need explicit encouragement to engage in learning-centred practices including the achievement of feedback literacy [7, 10]. This applies not only to their learning while undertaking their health profession programme but also in their preparation for a career of lifelong learning, where critical thinking and reflective practice is required for good clinical practice and mandated by professional associations and registration bodies.

The training of health professions typically involves both academic and clinic-based learning also known as work-based learning in the literature. In work-based learning, learners are guided from novice to independent learner through situated learning experiences. This pivots around the early establishment of supportive, collaborative relationships between educator and learner and the mutual willingness to engage in meaningful action-oriented feedback dialogues [2, 13]. Concurrently, the learner is often required to draw up a personal learning plan with their educator to enable them to navigate the complexity of work-based learning by identifying clear goals towards its achievement. A study of physiotherapy students [14] highlighted the importance of collaboration in learning, identifying collaboration as the most common learning style. Furthermore, the more participatory learners achieved higher academic performances compared to those in the avoidant group. It is likely that similar collaborative dialogues between teacher and learner in early classroom-based modules could help foster early interest among learners in engaging in a learning rather than grade-oriented journey. This would enable learners embrace the idea of ‘assessment for learning’ and the concept of reflection ‘in’ and ‘on action’; core elements of the ethos of lifelong learning for healthcare professionals.

The authors’ university, and specifically the School of Allied Health (SAH) has been overtly successful in embedding feedback of assessed work into their programmes. As with others in higher education institutes [8], the SAH utilises a unidirectional communication approach where feedback on summative assessed work is delivered by individual teachers at the end of the module and posted on online module sites on the university intranet. From random checks of feedback downloads in SAH, it was apparent that the many students did not access or engage with modular feedback provided, a problem which is reflected internationally [9]. Thus, it appeared that feedback was having little impact on subsequent student academic performance [12]. We determined that a better approach promoting engagement with feedback and fostering feedback literacy was required.

### Aims

The overarching aim of this study was to identify a feasible, sustainable approach to improve feedback literacy in students on pre-qualifying health professional programmes in order to encourage learners to adopt a critical thinking rather than a grade-oriented approach to education and thus, prepare learners for a career of lifelong learning and reflection. The study was divided into two phases: phases 1 and 2. Specific aims were to:

### Phase 1

- Develop and pilot an integrated online feedback system for learning and growth in under-graduate and post-graduate health professional programmes in SAH;
- Encourage learner use of individualised and integrated module feedback for learning and growth;
- Investigate the learner and teacher experience of integrated module feedback for learning and growth, and;

### Phase 2

- Resulting from the above, outline a best way forward to embed good feedback literacy practices into the programmes.

## Methodology

This mixed-methods study employed a constructivist approach which seeks transformative change through a participatory process. A constructivist method is increasingly employed in feedback studies [8, 15, 16], where stakeholder perspectives feed forward into project development and the creation of new understandings and impactful outcomes [17]. Such an approach may involve multiple steps including collecting and analysing data, re-engineering the research plan and taking of informed action. It is similar to action research in that it is a form of reflective enquiry undertaken by participants regarding specified practices.

In our study the participants were SAH teachers, SAH learners, the core research team (practice education and academic members of staff respectively) and the external advisory group of teaching and learning experts. The initial study action, developed based on the experience of the research team, knowledge of the literature in the area, and the principle of utilising integrated feedback to improve feedback literacy, was a pilot online intervention. As with all research involving iterative phases, monitoring of, feedback on, and collaborative discussion occurred after the roll out of phase one leading to the development of phase two. Barton et al. [8] refer to this process as “where collaborative wisdom and sharing of information informs the next cycle”. Thus, two phases of research were conducted involving the piloting of an online integrated feedback tool and the development of a suite of modular feedback components.

### Guiding pedagogical principles

- The development of feedback literacy through integration of all feedback received by a learner across all assessed work. Integration of all feedback ensures that core and repeated messages regarding the learner’s performance become more evident to the learner and thus the learner can better act to improve their performance in subsequent tasks.
- That students should be encouraged to be active learners [15] i.e. to self-evaluate their feedback across all modules and incrementally develop autonomy or self-regulation [8, 15, 18] regarding their identification, utilisation and application of core feedback learning points.
- That the institutions systems and practices should be utilised to operationalise the pilot programme and that this would contribute to programme sustainability. These include a functioning academic advisor system (each student is allocated an individual advisor), a culture of providing feedback for all assessed work and online module sites which students are familiar with for accessing learning resources and feedback.
- That feedback literacy would be developed through collaboration. Reinforcement of the learner’s efforts at identifying core learning points from their integrated feedback would be facilitated through a two-way teacher-learner discourse [15, 19] with their academic advisors (teachers) thus assisting supported reflection and helping the learners understand and act upon the integrated feedback.

### Context

This study was situated in the School of Allied Health (SAH) at the University of Limerick, Ireland, consisting of four allied health disciplines (human nutrition and dietetics, occupational therapy, physiotherapy, speech and language therapy) and five professional qualification programmes (one undergraduate, four post-graduate). At any one time, SAH has 300 professional qualification learners enrolled on its books. SAH has a core ethos of inter-professional education (IPE) with a number of modules specifically formulated using IPE principles and the remaining designed and delivered as discipline specific. SAH also has a stated commitment to learner engagement and the use of feedback for formative and assessed work. Thus, learners expect to receive feedback on their performance. Additionally, an academic advisee system has been operational for over a decade with all learners assigned an academic advisor in their first semester. Thus, this study was operationalised using systems already in place, where feedback was already provided as standard, in an organisation where the ethos promoted learner and teacher engagement with feedback practices. Within the institution, the study was supported by the Centre for Transformative Learning, the Head of SAH and respective programme directors.

### Software

To facilitate learners’ integration of feedback from assessed work, the authors employed an online feedback software system (FEATS) enabling learners to upload and integrate feedback from individual modules. A review of hard and electronic feedback systems was conducted prior to selecting FEATS which was originally developed by Winstone and Carless [20] using a constructivist approach between the Teaching and Learning Department at the University of Surrey and their students. FEATS requires learners to upload modular feedback at the end of each semester to a confidential personalised folder. Learners are required to categorise this feedback using predetermined themes for example, academic writing. Once students had registered and uploaded modular feedback, the FEATS system used this categorised information to enable automatic visualisation of each student’s academic strengths and weaknesses based on the totality of their feedback. See Additional file 1: Appendix 1 for an example.

### Participants

Two primary stakeholder groups participated in phase 1. All first-year learners in the under-graduate and post-graduate professional qualification programmes were eligible to participate (*n* = 123). Contact with the five cohorts was initially made in the first semester to provide training and normalise feedback literacy from study commencement. All SAH teachers who were academic advisors were invited to participate (*n* = 36). The core research team members (AOC, AMcC) were also teachers and thus available for advisee meetings. However to prevent bias, the authors did not act as study participants. In phase 2 convenience sampling was used to recruit academic advisors and student representatives from each of the respective programmes. Additionally, two international educationalists were recruited to provide further expertise from an educational perspective.

### Protocol

#### Phase 1 - learners

- Introduction to the project and the online system for uploading feedback (FEATS) during the first semester was provided in a selected inter-professional education module. Information sheets and consent forms were subsequently distributed by the module leader.
- At the end of the first semester once grades and feedback were released, FEATS was made available to all first-year learners registered on health professional programmes via an online link sent by email.
- Learners registered their FEATS account and uploaded modular feedback to a confidential personalised folder in order to categorise this feedback under pre-determined themes describing the specific sentiment of the feedback. These categories were determined based on a survey at SAH where teachers (both practice-based and academic) were asked to provide a list of the top three feedback issues on which they commonly provided feedback.
- Once feedback was categorised by the student, the FEATS system used this categorised information to enable automatic visualisation of each learners’ academic strengths and weaknesses. (Additional file 1: Appendix 1).
- Learners were asked to reflect on synthesised feedback and develop a learning action plan based on identified strengths and weaknesses thus providing a targeted approach towards improving academic performance.
- Drop-in tutorials/help clinics were offered to learners by the research team throughout to help navigate the online system; two reminders were sent to encourage engagement with advisors. Links to resources on how to use the online FEATS system were provided for students on a dedicated site on the universities online learning platform.
- Once learners had completed the uploading of feedback online they were encouraged to meet with their academic advisors to review their integrated feedback and discuss learning action plans.

#### Phase 1 –academic advisors (teachers)

- The study was introduced to academic advisors during two SAH team meetings in the semester preceding the launch of FEATS. Study information sheets were subsequently emailed to all advisors.
- Additional information and training sessions were offered to ensure all advisors were familiar with the project. Two reminders were sent to encourage engagement with learners regarding feedback during advisee meetings.

#### Phase 2 – development of feedback module

**Fig. 1:**
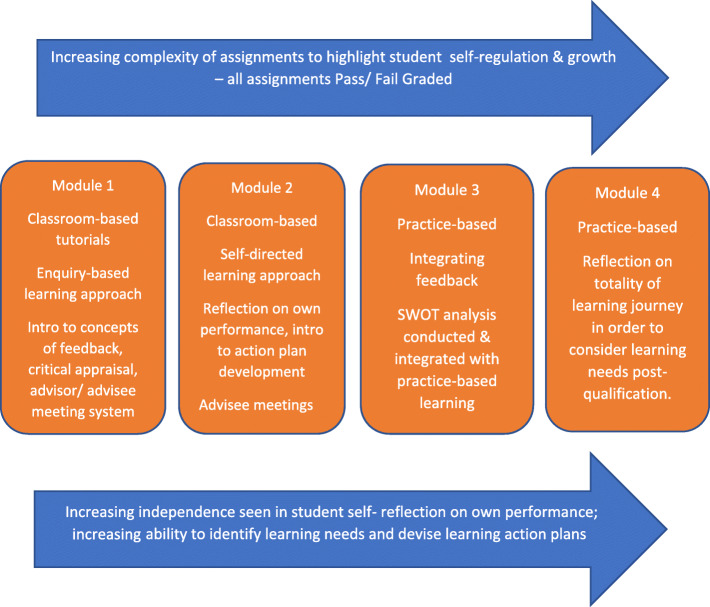
Summarised version of suite of modules

Phase 2 evolved from stakeholder feedback and the authors’ iterative reflections on progress with phase one. When it became clear that the online feedback system was not presenting a sustainable method of developing feedback literacy, the authors considered what factors were contributing to firstly, poorer than anticipated engagement among teachers and learners and, secondly the decline in learner engagement when developing learning action plans. It was acknowledged that the perceived demands may have been too high for entry-level learners (albeit mainly post-graduate) in terms of their ability to independently self-appraise feedback and develop action plans specific to weaknesses highlighted. This was reinforced by stakeholder feedback and led to a revised research plan. The revised research plan focused specifically on the development of a suite of modular components (see Fig. 1 (summarised detail) and Additional file 1: Appendix 2 (full detail)) designed for potential integration into SAH modules or as stand-alone modules depending on stakeholder feedback. The suite of modular components aimed to address engagement challenges with a module straddling each year of respective programmes to nurture a skillset of critical thinking and evaluative skills which would grow as learners progressed through their academic programme.

#### Ethics

Ethical approval was granted by the University of Limerick Education & Health Sciences Faculty Research Ethics Committee (2019_12_19_EHS).

##### Data analysis

Data collection and data analysis took the following forms:

1. FEATS category development: Data provided by staff regarding the most consistent feedback themes provided to students were analysed using a content analysis approach. An initial trawl through the data removed duplications. Data were grouped by theme using an iterative approach by the two researchers (AOC, AMcC) and synonyms developed to ensure transparency between all categories so that students could quickly choose the category their feedback aligned with.
2. FEATS usage: To determine user uptake a follow up survey of FEATS usage and stakeholder experience was disseminated to learners via student email addresses. Three core questions were asked: whether learners had launched FEATS, how easy it was to launch and whether learners categorised their feedback using the categories provided. Descriptive statistics were employed to analyse the data.
3. Stakeholder experience of FEATS: A follow-up survey was conducted with learners and advisors to explore perceptions of FEATS and the feedback process. Questions focussed on learner interaction with FEATS, perceived ease of use, perceived value, and engagement with academic advisors regarding the development of learning action plans. The survey was disseminated via survey link using internal email systems. Descriptive statistics were employed to analyse the data.
4. Perspectives on proposed suite of module components: Participants (academic advisors, student representatives and international educational experts) were asked to review the proposal (Fig. 1 (summarised) Additional file 1: Appendix 2 (full detail) and provide feedback via 1:1 online interview or hard copy. A question guide was developed (Table 1). Interviews were audio recorded and transcribed verbatim. Transcripts were analysed using thematic analysis [21]. The first author (AOC) analysed all transcript data. The second author (AMcC) read one interview transcript from each of the stakeholder groups to verify key themes. Following this both authors discussed the data and agreed main themes.

**Table 1:** Question Guide

**Question schedule for teachers/ learners and educational experts** **Introduction:**	
You have had a chance to look over the proposed module which aims to………	
Let me just re-summarise what it looks like…….	
**General Questions:**	
What are your first thoughts regarding the overall module in terms of meeting the demand for student support?	
What aspects of the module do you think will be attractive to students/ staff?	
What aspects do you think will be unattractive or challenging to students/ staff?	
Have you any recommendation for how you think this could be addressed?	
What do you think of the credit value of the module?	
What do you think of the method of assessment?	
What do you think of the increasing difficulty of tasks?	
What do you consider to be the best selling point for this module in order to maximise buy-in with students/ staff?	
**Staff only:** Do you think we will have difficulties with implementing this module in relation to current academic regulations and curricula and if what specifically?	
In your opinion is it in the institutions and students interests to be designing and planning modules such as this which focus on learning for growth?	
**Conclusion:** in summary we have discussed a,b,c… are there any other ideas or thoughts that we have not mentioned, that you would like to mention now?	
**Close:** Thank you for your time.	

## Results

### FEATS category development

Sixteen categories were identified by the research team based on feedback from academic colleagues for the purpose of facilitating students’ categorisation of their modular feedback: Argument construction, critical thinking and evaluation, documentation, effective use of literature, methodological skills, presentation skills, clinical assessment skills, clinical reasoning skills, collaborative working skills, communication skills, consolidation skills, professionalism, referencing, reflection, self-management and, writing skills. Synonyms were developed for each of these categories for the purpose of clarity and ease of use by students when uploading and categorising their modular feedback.

### FEATS usage

A total of 67 learners launched their own personal FEATS system representing an uptake of 54%. Twenty-five learners responded to the survey - a response rate of 20%. See Table 2 for results.

**Table 2:** Learner responses to survey

	n	Responses (%)
Total Learners	123	
Launched FEATS	67 (54.5%)	
Completed survey	25 (20.4%)	
How easy was it to launch?	18	
*Extremely / somewhat easy*	12	66
*Neither easy nor hard*	2	11
*Somewhat /very difficult*	4	22
Did you categorise your feedback under the headings provided	18	
*Yes*	12	67
*No*	6	33
Did you find the categories appropriate for your needs?	18	
*Yes*	8	44
*No*	10	56
Did you use the tool to develop an action plan?	18	
*Yes*	2	11
*No*	16	89
Rate your satisfaction with the resources provided via links	2	8
*Somewhat satisfied*	1	50
*Neither satisfied not dissatisfied*	1	50
Did you engage with your advisor regarding your action plan?	18	72
*Yes*	3	17
*No*	15	83
Was the meeting with your advisor regarding feedback useful?	3	
*Extremely useful*	1	33
*Moderately useful*	2	66
Did this exercise encourage you to engage with your feedback?	15	
*Yes*	9	60
*No*	6	40
Would you avail of it again?	19	
*Yes*	8	42
*No*	11	58

### Stakeholder experience of FEATS

Two-thirds (66%) of survey responders reported FEATS was easy to use. Of those who launched the system, 67% categorised their feedback but only 11% went onto complete a learning action plan. Further, 83% did not engage with their advisor despite being encouraged to do so. Those who did engage reported the advisee meetings as useful. When asked if they would avail of the FEATS system again, 58% said they would not. Six additional comments were provided to explain responses. Some of these comments suggested no perceived need for a focus on feedback literacy:

*“I am aware of my strengths and weaknesses already”*

*“I was already aware of the areas I needed to address but it was good to see them visually”*

*“FEATS seems pretty useless in our field of study. There is no need to use it. I am a student of occupational therapy”*

Other suggested more clarity was required regarding the purpose of FEATS.

*“Not sure on the need for integrated feedback”*

*“Wasn’t sure what it was for”*

A final learner’s comment suggested improving feedback literacy was not a high priority:

*“It appeared very time consuming and a little convoluted. Current course work took priority over this”*

### Academic advisors

Twenty-seven advisors responded - a response rate of 75%. Table 3 highlights those responses.

**Table 3:** Teacher responses to survey

	n	Responses (%)
Total Advisors	36	
Responded to survey	27 (75%)	
Did you attend any of the training sessions?	26	
*Yes*	5	19
*No*	21	81
Did you encourage the use of FEATS with your students?	26	
*Yes*	17	65
*No*	9	35
Did your students raise any questions about FEATS/ feedback during your sessions?		
*Yes*	5	19
*No*	21	81
What was the nature of the query?	5	
*Understanding the feedback provided by teachers*	1	20
*Locating feedback*		
*Synthesising feedback in categories*		
*Technical issues regarding software*	3	60
*Other*	1	20
Where you satisfied you had enough information to deal with the query?	5	
*Yes*	1	20
*No*	4	80
Based on your interact with students, do you think they benefitted?	26	
*Yes*		60
*No*		40

Of those responding to the survey, one-fifth (19%) had attended training and information sessions. Further, 65% reported encouraging learners to engage with FEATS. One-fifth (19%) reported learners had asked questions regarding feedback at advisee meetings. Most queries related to technical issues regarding software and 80% reported they had insufficient knowledge to deal with such queries. Notwithstanding, 60% believed the system was useful. Advisors provided three additional comments which reflect positivity about the pilot intervention and engaging learners in the area of feedback literacy.

*“Students can see how the system will be useful when they do clinical placements”*

*“I think students who have engaged have a greater appreciation of the value of feedback and an understanding of how to adapt feedback from one assignment to other modules and assignments”*

*“They have more awareness about feedback and it has created more of a willingness to proactively engage with it”*

### Perspectives on a suite of module components to promote learning and growth

In total seven students, six teachers and two international educationalists reviewed the proposal and provided feedback on the suite of modules presented. Three main themes were used to describe the data. These were: a) Short and long-term benefits, b) Stumbling blocks and c) Circumvention. Participants have been coded as L (learner), T (teacher) and E (expert) plus a number to delineate individual contributions.

#### Short and long-term benefits

Learners, teachers and educational experts overwhelmingly acknowledged the need for the development of feedback literacy as early as possible for university entrants.

*T3: It's a really good idea. Students need that support to learn how to reflect and how to use that to enhance learning. Why I really like the whole idea is that they would get support to reflect.*

*L2: It sounds really interesting. I like the idea of having that kind of agency in your own learning to take a bit of responsibility for it.*

E1: *This targets highly relevant competencies. It aims at making relevant knowledge, skills and attitudes explicit – which is an essential first step in competency development –*

Both stakeholder groups cited short- and long-term benefits to introducing feedback literacy as early as possible in each programme. Short-term benefits related to the potential improvement that could be realised in student’s academic performance in the short-term future.

*T2: The advantages short-term would be a selfish thing; how to get a better grade; how to improve academic performance.*

*L4: It's a skill that we need. To be able to critically reflect on your own learning is actually something you'll always need to be able to do. This would make it easier.*

*E1: I see the short-term benefits as those which provide explicit attention to specific, student ownership of learning trajectories, personalized feedback and opportunities to build long-term collaborative relationships as well as having conversations about the programme, the profession and what it takes to become a professional.*

Long-term benefits described by both stakeholders related to the requirement of these skills post-qualification when engaging in professional development activities, interview and curriculum vitae preparation.

*T4: They will need this for interviews and for long-term CPD. Examples of reflection will be expected. So short term- benefits - exams, long term benefits - CPD and interviews in the future.*

*L1: It would definitely motivate me to do it and it seems like a really good idea. It would benefit us definitely, it will help your CV.*

*E2: I really like the way in which the ethos of the model links feedback to students’ development as professionals, and how they will need to reflect and engage with feedback in professional practice. I think the connection between feedback processes and professional practice will enhance the perceived value to students.*

Thus, both stakeholder groups agreed that the unique selling point was the multiple benefits that development of feedback literacy would provide to students.

#### Stumbling blocks

Despite the positivity expressed, both stakeholder groups also conveyed concerns regarding implementation of the proposed suite of modular components. Learners for example, were concerned that their workload, already perceived as substantial, could be further exacerbated by adding a new suite of modules with extra assignments which they perceived could make it difficult for them to meet academic demands.

*L3: Maybe the timing of when those assignments would be required is probably one of the main things. Workload wise, that's the only thing I could see people complaining about, the pressure.*

Teachers also expressed concern regarding a potential increase in workload for themselves as well as students. Issues regarding module leadership and ownership of the proposed modules were highlighted, and how this might affect other academic roles in what was perceived to be an already overloaded curriculum.

*T1: Anything that involves change and increasing workload. And getting the time to conceptually get your head around it. That's something that's maybe not easy for everyone to do. Especially if somebody has a job that's around teaching and research, and they're thinking long-term about developing the research side of their role, then teaching becomes secondary.*

*T6: The workload issue is still a challenge. It seems like there will be more advisee meetings and longer meetings. I'm not saying that's like a stopping point, because obviously it's worthwhile, but it is definitely a challenge for students and I would say as well from a staff point of view.*

Educational experts also provided insight into aspects they felt could present as challenges to the success of the proposed modules and which could influence buy-in from both student and staff as stakeholders:

*E1: I believe that one of the major pitfalls in this project will be that it is perceived as yet another tick box exercise by both students and teachers. The biggest challenge will be to let students and teachers experience that the learning activities offered are essential and meaningful in the context of professional development (and not just something they have to do to obtain credits).*

*E2: Sometimes pass/fail assessments can be perceived negatively by students and it can promote an instrumental approach to just doing what is necessary to pass.*

Many concerns were voiced regarding teachers experience with providing this type of advisory role within their current workload, with many suggesting they either did not have the skills for it, or that it would take away from other roles within their academic remit that they cared more about.

*T5: It assumes that the person who is the advisor is experienced in feedback so you'll need to train some people.*

T2*: I wonder would there be too much room for variation in effort across academic staff? You can train them all you like, but if they want to engage they engage. If they don't, they won't.*

This was also alluded to by one educational expert who suggested that time and resources could be a significant factor in the success of the initiative

*E1: Teaching and learning activities that are perceived as irrelevant or not applicable to what students perceive to be core learning activities will challenge this initiative. Also resources (staff time, training and coaching of students) will need to be considered.*

#### Circumvention

It was understood by the authors that close consideration was required regarding the location of proposed modular content (i.e. embedded or standalone) and transparency regarding staff accountability for the achievement of the proposed learning outcomes throughout the programme.

*T2: If it was in a dedicated module, then one person providing a consistent message and standardization, that would be good in one way. But there could be a lot of variation in effort and interest if embedded in several modules.*

Almost all stakeholders believed that after the perceived failure in the first phase of the study to develop feedback literacy independent of the educational curriculum, the best way forward would be to provide the content in a module or suite of modules and award it a small credit value, for stakeholders to earnestly engage with it, yet at the same time minimise impact on workload while still achieving the potential gains.

*L3: I hate to say add more assignments to an already overloaded caseload but if you want to get student engagement then it is probably a good idea that it has some kind of value to students.*

*T3: If there was some way that we could work it so that we're not repeating what has already been done. We could embed it in some of these existing modules, then we could just add an assignment around the skill.*

*E1: It is essential to ensure that all teaching and learning activities are well-embedded in the curriculum. . For many learners, the main focus is on “passing the test” and figuring out how to proceed through the programme as quickly and smoothly as possible. This is understandable, but I think we should have ongoing conversations with students about what it is to be a competent professional and to become one, to start internalization of goals relevant for life-long learning, from day one.*

Notwithstanding, it was considered reasonable by all groups, that there should be specific assignments attached to feedback literacy content and these should be considered as part of the overall module grade. Both groups recommended the use of a pass/ fail grading system for assignments linked with the proposed suite of modules as it would facilitate the prioritisation of skill development over competition for grades.

*T6: Pass-fail is reasonable. You know, given that really what you want them to do is engage with the task…. you want them to do it and think about it.*

*L1: Yeah, pass/ fail I think. Some people could put in a huge amount of effort and some people could be done in five minutes and that's absolutely fine too as long as you can demonstrate that you've achieved the outcome.*

## Discussion

### Engagement

This study set out to pilot a method of promoting feedback literacy among health professional learners in their first academic year using a stakeholder-informed constructivist approach. The overarching aim in developing this initiative was to foster feedback literacy through the integration of all modular feedback from assessed work. It was anticipated that this would feed forward to future academic performance and work-based placements, thus optimising learners’ transition from academia to the healthcare workforce. Through evaluation of outcomes and stakeholder experience, the design transitioned from a learner-led online platform, to a teacher-supported suite of modular components intended to straddle each year of respective programmes. This was necessary when it became clear that the requirement to complete independent learning plans was too onerous for first year learners. Reasons cited by learners for early disengagement related to academic workload, time commitments and limited relevance to future academic work. These findings are commonly reflected in current literature [9, 22] - learners often see their educational journey as the completion of a series of exercises rather than a pathway towards skills development. Another factor which may have impacted on student engagement in the first phase of the study was that participation was voluntary and not rewarded with credits or grades. It is well-known that learners are grade oriented [12]. Therefore, students may have considered this exercise to be of limited academic and personal value, a phenomenon highlighted in previous research [2, 23].

Lack of engagement was not only reflected in the learner cohorts but also in teachers. Uptake of training in Phase 1 was limited despite continued offers of support by the research team including drop-in clinics and training workshops for the duration of the implementation phase. No additional workload was required of teachers as facilitation of feedback literacy was embedded into already occurring advisee meetings. Despite this, engagement and support of students in this phase of the study was limited. Thus, it can be concluded that the development of feedback literacy is a multi-stakeholder issue, the failures of which cannot be solely laid on learners’ shoulders nor explained by claims of grade-oriented students. It requires critical investment from both teachers and learners to be successful and for teachers to commit to facilitating learners not only on their short -term educational journey but nurturing a life-long learning ethos in students. For feedback literacy to bear fruit, it is imperative that both learners and teachers collaboratively engage to reap benefits. This ultimately requires a shift in thinking on the part of students and teachers. This is reflected in the ethos of programme-based assessment, where assessment of student progress seeks to evaluate students’ achievement of programme outcomes rather than the traditional modular approach. This framework has been successfully employed among some health professional programmes in order to develop the learner through a focus on student growth and development and warrants broader consideration.

### Supporting feedback literacy going forward

Reflection on student experiences in Phase 1 led the authors to recall similar challenges in the work-based learning context among novice learners. The work-based learning experience is built upon the premise that the clinical supervisor and learner enter a supportive working relationship in order to realise learning outcomes. Over time, as learners become more confident in their ability and skills, the clinical supervisor gradually steps back as the learner gains independence in their self-appraisal skills and self-regulation of learning. The authors utilised this model as a guiding framework for the development of the a suite of modules. Similar to our findings, Bloxham and Campbell [24] found that their students were not experienced enough to know what was expected in order to initiate meaningful dialogue with their tutors. This highlights that facilitation is required to help learners, from the beginning of their academic programme, in order to embed it in all aspects of their learning journey.

All cohorts overwhelmingly agreed that the principles of the suite of modular components would pay dividends both in the short and long-term regarding student learning and academic achievement. Workload constraints, however, were highlighted by both stakeholder groups as a potential threat to the implementation of the proposal. This raises a clear requirement for a full review of current programmes being perceived by both the authors and teachers as a significant barrier to the immediacy with which the suite of modular components could be implemented. Ferrell [25] in a recent report described as “stubbornly resistant to change” the feedback terrain in higher education. It potentially reflects the prioritisation of knowledge/skill acquisition in the educational journey as opposed to a broader ethos focusing on the development of the learner. For feedback literacy to develop it must be embedded in the curriculum and embraced by both learners and teachers as an essential, rather than additional, part of professional degree requirements. This is not an impossibility as Barton et al. [8] demonstrated in their study, highlighting the importance of getting buy-in from all stakeholder groups and demonstrating for example that despite the increased time it takes for staff to engage with the feedback dialogue, all staff wanted to continue with it. They further suggested compromise was helpful in moving feedback literacy forward to balance the time requirements of teachers and learners. In their case reducing the number of overall assignments on programmes provided a means of encouraging stakeholder to engage in feedback activities. Further, while programmes remain grade and not learning focused, its impact and potential will remain muted.

## Conclusion

The development of feedback literacy among health professional learners in the early stages of their academic journey is crucial. The development of these skills affords an opportunity to develop openness in the learning relationship between student and teachers. This in turn may foster early development and embedding of critical thinking and self-appraisal skills, thus preparing students for lifelong learning in their professional careers. The transferability of such skills has been highlighted in the literature and by all stakeholder groups involved in this study who cite short-term academic gains and long-term benefits post-qualification as clear benefits. Finding a balance between introducing these skills at a time early enough to highlight their importance among university entrants is challenging. Further balance must be achieved between the workload required to achieve these skills and current programme demands for both teachers and learners. This must be weighed up carefully to optimise buy-in for students and staff. Ultimately a clearly supported trajectory is essential where students can see for themselves where the educational journey begins and where learning gained will take them. Notwithstanding the scope of the developmental work described in this study, teachers setting out to develop early feedback literacy skills in students should endeavour to initiate a structured meeting system in the first year of university programmes between academic staff and students to nurture openness and self-appraisal in a non-judgmental, constructive environment in order to facilitate self-regulation and growth.

### Limitations

The authors acknowledge the influence that COVID-19 may have had in terms of uptake of the online feedback system among students during the initial phase of the study and on recruitment for the initial survey. Notwithstanding the small sample size of same, the authors contend that findings would not have been significantly different had this study been conducted outside of COVID-19 times, evidenced by the alignment of our findings with current literature. While this study was conducted in a health professional context, the authors emphasise the transferability of findings from this study to other professional programmes delivered in higher education and strongly contend that the suite of modules developed in this study could serve as a framework for other professional programmes seeking to develop feedback literacy in similar contexts. Finally, as this suite of modules has not been implemented yet, based on the requirement for further organisational structure and review, it is unclear what the benefits to students will be. Nonetheless, it is anticipated that the module will deliver on the learning outcomes outlined having been strengthened by the co-constructivist methodology employed in the second phase where students and teachers were involved in further refining of the feedback initiative.

## Supplementary Information

**Additional file 1:.** Appendix 1: Sample of output of learners’ top skills and areas of improvement. Appendix 2 Information on Module Design for Phase 2.

## Data Availability

The datasets used and/or analysed during the current study are available from the corresponding author on reasonable request.
