# Supplementary material for: A feedback journey: employing a constructivist approach to the development of feedback literacy among health professional learners
Source: BMC Med Educ. 2021 Sep 10;21:486. doi: 10.1186/s12909-021-02914-2 (PMC8429041; doi:10.1186/s12909-021-02914-2)
Supplement: Supplementary file 1 — Additional file 1:. Appendix 1: Sample of output of learners’ top skills and areas of improvement. Appendix 2 Information on Module Design for Phase 2. [file 12909_2021_2914_MOESM1_ESM.docx]

Appendix 1: Sample of output of learners’ top skills and areas of improvement


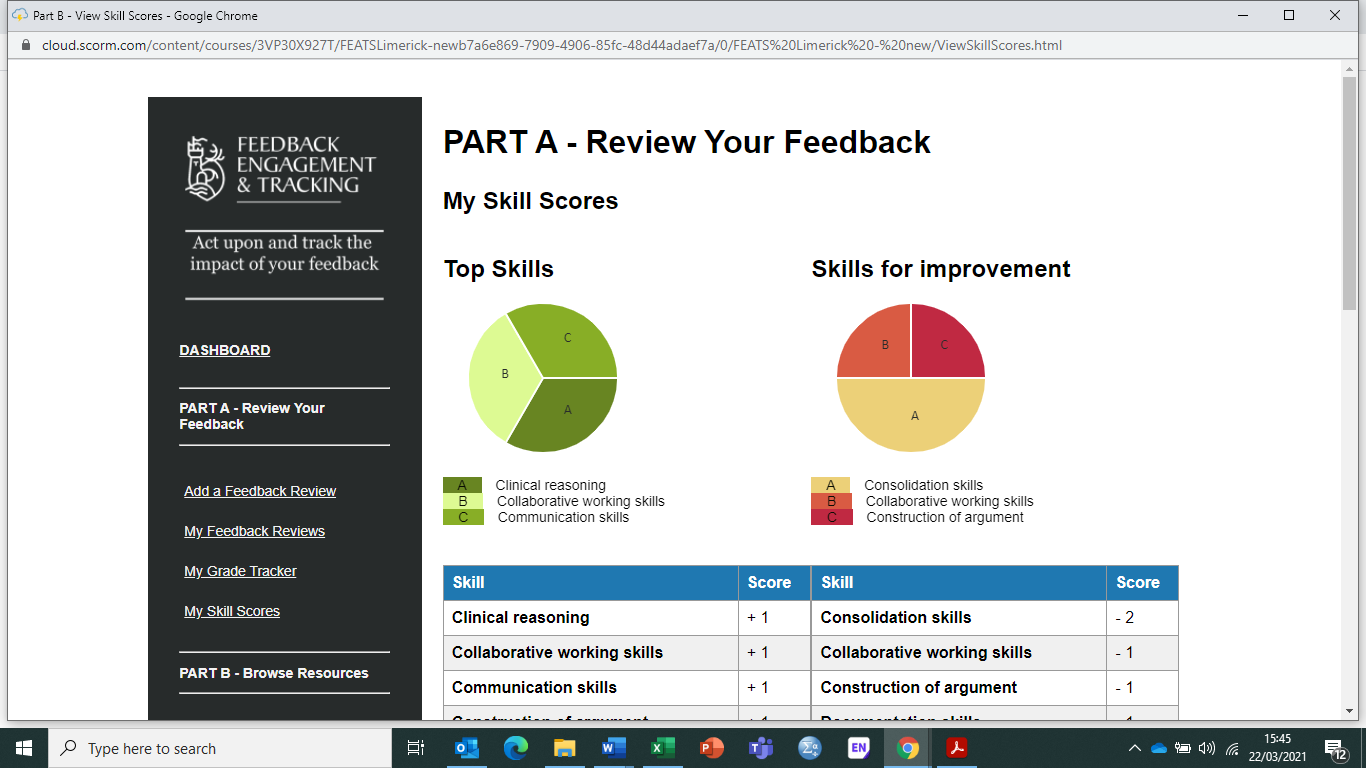


Appendix 2 Information on Module Design for Phase 2:


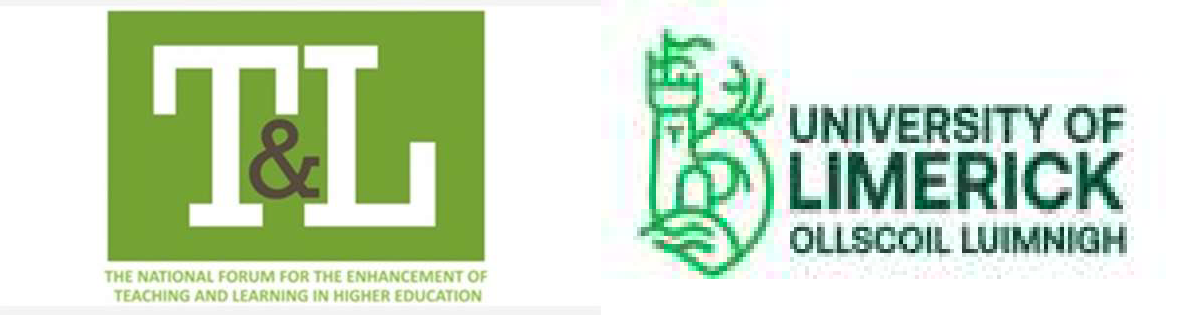


Integrated Feedback for Learning and Growth- iFLAG

# Dr. A. O’Connor & Dr. A.McCurtin

School of Allied Health, University of Limerick.

Introduction to iFLAG

The iFLAG study was initiated in 2020 following funding awarded by the National Forum for the Enhancement of Teaching and Learning, Ireland in 2019 to develop this aspect of teaching and learning across disciplines. The aim of iFLAG was to develop a sustainable means of facilitating students towards independence in the integration of academic feedback into their academic work for the purpose of improving academic performance. Additionally, a long term aim was to foster critical thinking, and self- reflection skills in health professional students early on in their educational trajectory, to build and develop a seamless transition from student to qualified health professional. Current evidence highlights that while a lot of time and effort is spent by academic staff in providing feedback to students, this feedback is frequently perceived as invaluable, irrelevant or received too late for students to be able to act on it in a timely manner. Therefore, the iFLAG study set out to promote the integration of academic feedback by encouraging students to understand feedback better in order to formulate learning action plans to improve their academic performance.

Initially, an online feedback system (FEATS) was implemented early 2020 which invited students to upload, categorise and synthesise academic feedback from their first semester. Students were then asked to develop a learning action plan for the next semester based on this feedback synthesis. Following a student evaluation survey, the research team acknowledged that students may not have been sufficiently equipped to develop the required learning action plan independently. Thus, the research study plan was reviewed and revised to propose a suite of modules which would straddle each year of allied health programmes, encouraging a collaborative learning relationship between students and their academic advisors in order to foster specific skills including critical thinking, self-appraisal, reflection and self-regulation of learning early in each programme in a more supportive manner for students. The use of an e-portfolio is proposed as a means of enabling students demonstrate achievement of the learning outcomes associated with the suite of iFLAG modules. The choice of e-portfolio is rationalised based on its use post-qualification as a means of recording continuing professional development for health professionals.

DEVELOPMENT OF IFLAG MODEL

SUITE OF iFLAG MODULES

*Background*

A series of modules, known as iFLAG modules, will straddle the 2 years of each graduate entry programme and years 3 and 4 of BSc programmes. These represent a series of hierarchical modules focused on the development of student learning and growth using modular feedback as the tool to structure learning. They are designed to facilitate students to become autonomous, critical, reflective life-long learners, who will use modular feedback from their assessed work to help them develop as learners in a sequential way. The overarching concept supporting these modules is that the educational journey is about developing the student to be an independent lifelong learning by the time they complete their programme, and to minimise the emphasis on learning for assessment and grades.

Universities need to commit to strategies that help students understand themselves and reach their fullest potential as learners, particularly those students who will need to employ critical thinking and life-long learning skills in their professional practice post qualification. The teaching and learning principles associated with these modules align with that of programme-based learning where the aim is to maximise learning, growth and development as a professional through formative feedback, collaborative discussion, and a gradual shift towards independence as a novice health professional

MODULE DEVELOPMENT AND DESIGN

*iFLAG 1 (semester 1)*

This module is an introduction to the iFLAG process and will cover topics such as feedback, integrated feedback, critical thinking, cognitive biases, lifelong learning, collaborative learning and the academic advisor system. It will be taught by group tutorials, online support and will employ an enquiry-based learning approach. This introductory module will prepare students for collaborative work with their academic advisors and the unfolding focus on autonomous learning to follow in the subsequent modules. There will be a formative assessment (a reflective exercise based on learning achieved for completed semester and planned for second semester). This will be M graded with iFLAG Module 2.

There are no prerequisite modules.

*iFLAG 2 (semester 2)*

Students will use the feedback provided on assessed academic work from Semester 1 as the basis of this module. With their academic advisors they will be asked to identify the strengths identified across all assessed work, and areas for development. An online system (FEATS) may be used for this purpose at a small cost to the university. This software allows students to upload and categorise their feedback and then provides an automatic visual representation of the students overall academic strengths and areas for improvement. In conjunction with their academic advisor the learner will consider the synthesised feedback and use it to inform the development and completion of an e-portfolio assignment. For this module, the student will be required to utilise their academic feedback to demonstrate achievement of one graduate attribute. This will form the basis of their assessed work for the semester. In meetings with their academic advisor, feedback will be discussed, in particular areas which are unclear to the student. Strengths will be reinforced and weaknesses identified, clarified and discussed. The assessed work will be marked on a PASS/FAIL basis and will be presented through the e-portfolio system. Prerequisite module: iFLAG Module 1.

*iFLAG 3 (semester 4)*

Students will continue their facilitation meetings with academic advisors. During these modules they will continue to use academic feedback from previous assessed work in Year 1 (academic and clinical modules) to develop an integrated SWOT (strengths, weakness, opportunities, threats) analysis. Two key tutorials will be provided in this module- one on integration of feedback and one on SWOT analyses. Online support will continue for the purpose of peer support and shared learning. Students will be asked to demonstrate achievement of a further graduate attribute as part of their assignment for this module (different to the one chosen for the previous academic semester), utilising the findings from their SWOT analysis in order to demonstrate achievement of the chosen attribute. This module will be M graded with iFLAG 4. Prerequisite modules: iFLAG2.

*iFLAG 4 (semester 5)*

During this module, students will reflect on their learning journey from Year 1 and 2 (Years 3 & 4 of BSc programmes) and use the totality of their feedback and their SWOT analysis to develop an action plan for lifelong learning post-qualification. This will be initiated by a process of reflection and self-appraisal regarding their perceptions on their own readiness for independent clinical practice. Following this reflection, they will develop their action plan for lifelong learning. They will receive 2 tutorials during this semester - one on Action plans and one on Lifelong learning. They will also receive a workshop on the use of portfolios for the purpose of documenting and demonstrating continuing professional development activities post qualification. They will have one meeting with their academic advisor to discuss the Action Plan. This will be uploaded and submitted for assessment via the e-portfolio system on SULIS. This will be marked on a PASS/FAIL basis. Prerequisite module: iFLAG3.

*iFLAG SUITE of MODULES*

*Aims*

- 1. To develop, critical, reflective life-long learners.
  2. To integrate academic feedback into academic and practice-based modules.
  3. To demonstrate achievement of graduate attributes.

*Objectives*

1. To support the use of feedback of assessed work to assist future learning
2. To encourage students to integrate feedback across modules to assist with ongoing and future learning
3. To enable students to understand factors which contribute to, and impact on their learning and growth
4. To enable students to develop considered, targeted, individualised learning action plans
5. To prepare students in the health professions to maintain a learning e-portfolio.

METHOD OF ASSESSMENT

Aligning with the principles of programmatic assessment, a Pass/Fail and M grading system will apply to the e-portfolio throughout each successive module. This is based on the need to encourage students to focus on the learning process and to engage in open discussion of their development as a self-directed learner and ultimately self-directed health professional post-qualification with their academic advisor. Achievement of learning outcomes will be guided, demonstrated and assessed through an e-portfolio where assignments will be designed around tasks which will enable the student to demonstrate achievement of graduate attributes using feedback from previously assessed work. These learning tasks and activities tasks will be uploaded to an online e-portfolio. Students will be encouraged to collaboratively discuss how best to demonstrate achievement of tasks with their academic advisor. It is envisaged that later in their educational programme, they will become independent in this activity and may only seek confirmation or minimal guidance from their academic advisor in order to design and deliver on the learning task.

In order to encourage and maximise student input and interaction in the suite of modules, each module will be assigned 5 ECTS. These may be offered as standalone modules, or alternatively, the 2 final modules may be embedded in practice education modules 3 &4 and form part of the assessed work for these modules. There is a possibility that IFLAG 1 could also be embedded in the Preparation for Practice Module in Year 1 Graduate Entry Programmes, and Year 2 BSc Physiotherapy programmes which may provide a more seamless introduction of the suite of modules into each programme. Assessed work in the first 2 modules may be graded as P/F/M-graded and will be submitted through an e- portfolio on SULIS. Assessed work if embedded in practice education modules will form a summative component
